# Fast and High-Resolution Neonatal Brain MRI Through Super-Resolution Reconstruction From Acquisitions With Variable Slice Selection Direction

**DOI:** 10.3389/fnins.2021.636268

**Published:** 2021-06-16

**Authors:** Yao Sui, Onur Afacan, Ali Gholipour, Simon K. Warfield

**Affiliations:** ^1^Computational Radiology Laboratory, Department of Radiology, Boston Children's Hospital, Boston, MA, United States; ^2^Harvard Medical School, Boston, MA, United States

**Keywords:** neonatal brain MRI, super-resolution, image reconstruction, anisotropic acquisition, isotropic reconstruction, fast imaging, spatial resolution, high-resolution MRI

## Abstract

The brain of neonates is small in comparison to adults. Imaging at typical resolutions such as one cubic mm incurs more partial voluming artifacts in a neonate than in an adult. The interpretation and analysis of MRI of the neonatal brain benefit from a reduction in partial volume averaging that can be achieved with high spatial resolution. Unfortunately, direct acquisition of high spatial resolution MRI is slow, which increases the potential for motion artifact, and suffers from reduced signal-to-noise ratio. The purpose of this study is thus that using super-resolution reconstruction in conjunction with fast imaging protocols to construct neonatal brain MRI images at a suitable signal-to-noise ratio and with higher spatial resolution than can be practically obtained by direct Fourier encoding. We achieved high quality brain MRI at a spatial resolution of isotropic 0.4 mm with 6 min of imaging time, using super-resolution reconstruction from three short duration scans with variable directions of slice selection. Motion compensation was achieved by aligning the three short duration scans together. We applied this technique to 20 newborns and assessed the quality of the images we reconstructed. Experiments show that our approach to super-resolution reconstruction achieved considerable improvement in spatial resolution and signal-to-noise ratio, while, in parallel, substantially reduced scan times, as compared to direct high-resolution acquisitions. The experimental results demonstrate that our approach allowed for fast and high-quality neonatal brain MRI for both scientific research and clinical studies.

## 1. Introduction

Magnetic resonance imaging (MRI), as a noninvasive neuroimaging method, has revolutionized our knowledge over the past 20 years in understanding the human brain. Imaging for neonates and infants enables studying brain developments and neurodevelopmental disorders from early stages, which is crucially important to both scientific research and clinical studies (Weisenfeld and Warfield, 2009; Giampietri et al., 2015; Mongerson et al., 2019; Tortora et al., 2019; Ding et al., 2020). However, it is challenging to precisely delineate the anatomical structures of the brain of neonates due to the small size of brain tissues in comparison to adults (Dubois et al., 2020). The spatial resolution is thus a critical factor in neonatal brain MRI. The typically used spatial resolutions in current clinical MRI practices, such as 3D imaging at isotropic 1 mm and 2D imaging with 0.5 mm in-plane resolution and 2 mm slice thickness, unfortunately, incur more partial voluming artifacts in neonates than in adults.

The interpretation and analysis of MRI of the neonatal brain benefit from a reduction in partial volume effect by increasing spatial resolution (Makropoulos et al., 2018; Dubois et al., 2019). Unfortunately, direct high-resolution (HR) MRI acquisition is time consuming and costly, and suffers from reduced signal-to-noise ratio (SNR). The long MRI scan for high spatial resolution potentially causes motion artifacts (Afacan et al., 2016). It is more prominent to the neonates who cannot be sedated, e.g., in a scan for the purpose of scientific research where sedation is typically unavailable. Also, even in scans where sedation is enabled to avoid subject motion, e.g., in a clinical scan, the long MRI scan for high spatial resolution leads to a substantial reduction in SNR, which in turn, increases the difficulty in distinguishing the signal of interest from noise. The underlying principle, from the imaging physics perspective, is that the reduced voxel size raises a reduction in the amount of signal received by the individual voxels. Consequently, the acquisition of short duration is critically important to neonatal MRI. The limitations of direct HR acquisition, therefore, necessitate the development of the methods that allow for imaging for neonates at high spatial resolution and high SNR, while in parallel, with short scan duration.

Current methods address the above limitations with a number of techniques, including parallel imaging (Pruessmann et al., 1999; Griswold et al., 2002), shifting to the ultra high field (7T) MRI (Annink et al., 2020), and super-resolution reconstruction (SRR) (Plenge et al., 2012). Parallel imaging and 7T MRI rely on hardware and imaging platforms, such as high density phased array receive coils and appropriate pulse sequence modification. In contrast, SRR, as a post-acquisition processing method, is performed on the acquired data that is in general of low spatial resolution and high SNR. Therefore, SRR is not subject to these limitations in hardware and platforms.

SRR originated in Tsai and Huang (1984) and was used for improving the quality of natural images. Fiat (2001) introduced SRR to MRI. It was showed in Scheffler (2002), and Peled and Yeshurun (2002) that SRR is unable to enhance the in-plane resolution of a 2D MRI or the resolution of a true 3D acquisition due to the Fourier encoding scheme. Also, it was demonstrated in Greenspan et al. (2002) that SRR is effective to improve the through-plane resolution of acquisitions of 2D slice stacks since the slices are individually Fourier encoded. Consequently, current SRR methods are designed to reduce the slice thickness of 2D slice stacks. Combining multiple low-resolution (LR) scans with different orientations was leveraged in Shilling et al. (2009), and then this framework was extended in Poot et al. (2010) to perform SRR with arbitrary image orientations and translations. SRR was quantitatively assessed and experimentally demonstrated in Plenge et al. (2012) to allow for a trade-off between spatial resolution, SNR, and acquisition time. Extensive SRR methods have recently been developed to improve MRI quality with a various of techniques (Gholipour et al., 2010a,b, 2015; Rousseau et al., 2010; Murgasova et al., 2012; Scherrer et al., 2012, 2015; Van Reeth et al., 2012; Kainz et al., 2015; Dalca et al., 2019; Sui et al., 2019, 2020).

SRR algorithms can mainly be classified as either a learning-based or a model-based method. Learning-based SRR summarizes the patterns mapping between LR and HR images over HR training data sets. Deep learning-based SRR has recently gained significant interest (Chaudhari et al., 2018; Chen et al., 2018; Zhao et al., 2019; Cherukuri et al., 2020; Wang et al., 2020; Xue et al., 2020). However, these methods require a large number of HR MRI acquisitions as the training data sets to learn the SRR model. The quality of the training data sets directly determines the quality of SRR. As discussed above, however, it is practically challenging to acquire HR data sets. Therefore, model-based SRR is commonly used in practice. Model-based SRR relies on an MRI acquisition model, from which an inverse problem is derived. As SRR estimates the super-resolved slices from much fewer acquired slices, the inverse problem is severely ill-posed. Prior knowledge, also known as regularization, is typically incorporated to separate the optimal estimate from the infinitely many solutions to the inverse problem. State-of-the-art priors include total variation (TV) (Plenge et al., 2012; Shi et al., 2015; Tourbier et al., 2015), non-local mean (Manjón et al., 2010), and gradient guidance Sui et al. (2019, 2020).

In this work, we developed a methodology for SRR based on the gradient guidance regularization method (Sui et al., 2019). It allows for high spatial resolution MRI with high SNR, excellent contrast-to-noise ratio (CNR), and reduced scan time, in comparison to direct HR acquisition. We achieved high quality brain MRI at a spatial resolution of isotropic 0.4 mm with 6 min of imaging time, using SRR from three short duration scans with variable directions of slice selection. Motion compensation is achieved by aligning the three short duration scans together. Our technique is thus suitable for use in a setting where direct HR acquisition is impractical. We applied this technique to 20 newborns and assessed the quality of the images we reconstructed. Experiments show that our SRR approach achieved considerable improvement in spatial resolution and SNR, while, in parallel, substantially reduced scan time, as compared to direct HR acquisition. The experimental results demonstrate that our approach allows for fast and high-quality neonatal brain MRI for both scientific research and clinical studies.

The novelty of this work is four-fold: (1) We take advantage of undersampling which allows us to form three undersampled neonatal scans with reduced acquisition time; (2) We encode the HR k-space data with three rapid undersampled observations of the HR k-space data convolved with a spatially oriented low-pass filter (being oriented axial, coronal, and sagittal). The estimation of the HR image from the undersampled observations is formulated as a deconvolution reconstruction problem; (3) The deconvolution reconstruction benefits from priors on edge position, which are easy to obtain and accurate in our setting; and (4) We apply our technique to neonatal brain MRI and achieve high quality images with reduced acquisition time.

## 2. Materials and Methods

The purpose of our approach is to construct neonatal brain MRI images at isotropic high spatial resolution and high SNR with reduced acquisition time for both scientific research and clinical studies. We develop an SRR technique that can reconstruct isotropic HR images from multiple anisotropic acquisitions with variable directions in slice selection. To assess our approach, we simulated an MPRAGE data set based on images at an ultra high resolution of isotropic 250 μm and acquired 60 T2 FSE images from 20 newborns on a Siemens 3T scanner. In this section, we present the theory and algorithm used in our approach, the detailed descriptions of our data sets, the criteria used in the assessments, and the experimental designs, respectively.

### 2.1. Neonatal MRI Acquisition Strategy

As SRR is effective in enhancing the through-plane resolution of 2D slice stacks, we acquire the images with large matrix size and thick slices. The large matrix size ensures the in-plane high resolution while the use of thick slices enables short scan duration and high SNR. However, the thicker the slices, the more severe the partial volume effect, and thus the more difficult the super-resolution. To this end, we acquire multiple LR images to facilitate SRR, where an increased number of slices are acquired. However, the total acquisition time is increased accordingly due to the increased number of scans. Fortunately, we can employ fast imaging techniques to accelerate the scans, such as fast spin echo (FSE) imaging. For images that yield long repetition time (TR), such as T_2_-weighted images, the FSE technique can significantly reduce the scan duration by *n*_*ETL*_ times with an echo train length (ETL) of *n*_*ETL*_ that typically ranges from 4 to 32 in clinical routines.

The goal of SRR is to estimate the missing signal in k-space based on the sampled k-space data. Our approach performs the estimation in the spatial image domain, which relates to the k-space data through Fourier transforms. Variable slice selection directions are incorporated in the acquisitions of the LR scans, where each LR scan contains a certain amount of k-space data in the slice selection direction. Consequently, the LR scan set comprises the spatial frequencies in different directions in the 3D frequency spectrum space. By combining multiple such LR scans, the difficulty of the SRR is thus reduced as an increased amount of k-space data is sampled. Although the slice selection directions and the number of the LR scans can be arbitrary, orthogonal (axial, coronal, and sagittal) acquisitions typically achieved a trade-off between acquisition time and SRR performance, since the acquired data yields the three complementary imaging planes.

We acquire three T2 FSE images from each neonate with variable directions in slice selection, which are typically carried out in three complementary planes (axial, coronal, and sagittal), and perform SRR to form an isotropic HR image. We set the parameters according to the scan time:

(1) $$\begin{array}{r} T≃TR\cdot \left\lceil \frac{FoV_{p}}{S_{p}\cdot f_{acc}\cdot ETL}\right\rceil \cdot N_{NEX}, \end{array}$$

where *FoV*_*p*_ denotes the Field of view (FoV) in the phase encoding direction, *S*_*p*_ denotes the voxel size in the phase encoding direction, *f*_*acc*_ is the acceleration factor of parallel imaging, *ETL* is the echo train length, *N*_*NEX*_ is the number of excitations, and *x* returns the smallest integer that is >*x*. We recommend that *FoV*_*p*_ ranges from 120 to 150 mm to fit the head size of the subject. *S*_*p*_ is kept at 0.39 mm. GRAPPA parallel imaging is leveraged with an acceleration factor of 2. Averaging is not considered in our fast imaging protocol, so *N*_*NEX*_ = 1. We recommend using *ETL* between 16 and 21 for fast scans of high quality. *TR* is typically set over 10 s depending on the number of slices required as well as the head size of the subject. We typically acquire 60–80 slices per image, and the slice thickness is fixed at 2 mm. It takes <2 min with our fast imaging protocol to acquire a T2 FSE image, i.e., *T* ≤ 120 s. For the largest value of FOV, i.e., *FoV*_*p*_ = 150 mm, with an *ETL*=21, it allows a *TR* ≤ 13.1 s according to (1), which is a sufficiently high value for TR. Consequently, our protocol can ensure less than two minutes of imaging time to acquire a T2 FSE image at the in-plane resolution of 0.39 mm for a neonate. Besides the parameters related to the scan time, we set TE = 93 ms, flip angle = 160°, and echo spacing = 9.8 ms. We use an interleaved acquisition mode, with which an even-first ascending slice order with an interleave factor of 2 is incorporated, i.e., the slice order is [2:2:*N*, 1:2:*N*−1] for an image with *N* slices. The HR image is reconstructed at the resolution of isotropic 0.39 mm, which is sufficiently high for the interpretation and analysis of the anatomical structures of the neonatal brain in clinical practices.

### 2.2. Neonatal MRI Reconstruction

We leverage the gradient guidance regularized SRR algorithm (Sui et al., 2019) to reconstruct the neonatal MRI images^1^. Given *n* acquired LR images ${\left\{Y\right\}}_{k=1}^{n}$, the forward model that describes the MRI acquisition process can be found from the HR image X by

(2) $$\begin{array}{r} {\text{y}}_{k}={\text{D}}_{k}{\text{H}}_{k}{\text{T}}_{k}\text{x}+\varepsilon_{k},\;,k=1,2,3,\ldots ,n, \end{array}$$

where **y**_*k*_ and **x** are column vector form of Y_*k*_ and X, respectively; **T**_*k*_ denotes a coordinate transform of X in the 3D space; **H**_*k*_ denotes a blur kernel; **D**_*k*_ denotes a downsampling operation; and ε_*k*_ denotes the imaging noise.

The noise ε_*k*_ can be considered as additive and Gaussian when SNR>3 (Hansen and Kellman, 2015). Therefore, the noise in each acquisition can be independently formulated as an identical Gaussian distribution. The HR reconstruction **x** is consequently obtained by solving the inverse problem

(3) $$\begin{array}{r} \underset{\text{x}}{\min}\overset{n}{\underset{k=1}{\sum }}\|{\text{D}}_{k}{\text{H}}_{k}{\text{T}}_{k}\text{x}-{\text{y}}_{k}{\|}_{2}^{2}+\lambda \underset{s\in S}{\sum }\|\nabla_{s}\text{x}-{\text{g}}_{s}{\|}_{1}, \end{array}$$

where S indexes a set of spatial image gradients, **g**_*s*_ denotes the *s*-th component of the gradient guidance, ∇_*s*_**x** computes the *s*-th spatial gradient of **x**, which is calculated from the same orientation and the same scale as **g**_*s*_, and λ>0 is a weight parameter for the regularization term. The above minimization can be accomplished by a subgradient descent (Bertsekas, 1999) or a proximal gradient descent algorithm (Daubechies et al., 2003).

As the images are acquired fast, we consider that there is no intra-volume head motion during the acquisition. Therefore, the transform **T**_*k*_ in Equation (3) compensates for the misalignment between scans. We use a rigid body transform to represent the misalignment. Consequently, **T**_*k*_ is defined by the parameters of six degrees of freedom (three for rotation and three and translation). We first interpolate all the LR images to those of the same size and the same resolution as the HR image being reconstructed by using a third-order B-spline interpolation method. We set **T**_1_ to an identity transform and evaluate **T**_*k*_ for *k*>1 by aligning the *k*-th interpolated LR image to the first interpolated LR image. In the alignments, mutual information is leveraged to measure the similarity between the first and *k*-th images. We use the CRKIT^2^ to accomplish the image alignment.

The blur kernel **H**_*k*_ in Equation (3) is a spatial invariant operator. It raises the partial volume effect in the acquired image. As only the through-plane resolution is enhanced while the in-plane resolution is kept unchanged, we design the blur kernel as a low-pass filter in the slice selection direction, also known as the slice profile. In the MRI acquisition, each slice is excited by incorporating a selective gradient that is generated by the radio frequency (RF). Ideally, the slice profile is desired to be a boxcar function. This requires infinitely many frequencies to yield the RF, which are impossible to obtain in practice. It is crucial to appropriately approximate the slice profile in SRR as the approximation directly influences the accuracy of the forward model. In general, bell-curve profiles with wider bases and narrower central peaks are leveraged, and slice thickness is measured as the full width at half maximum (FWHM) signal intensity. Gaussian profiles are widely used in MRI reconstruction and have been demonstrated to be effective in SRR (Rousseau et al., 2005; Jiang et al., 2007; Gholipour et al., 2010a,b; Murgasova et al., 2012; Sui et al., 2019, 2020). Therefore, we approximate the slice profile by a Gaussian function with an FWHM equal to the slice thickness.

As the downsampling factor can be arbitrary, instead of an integer for natural images, it is inconvenient to perform the downsampling in the image domain. Consequently, the downsampling operator **D**_*k*_ in Equation (3) is implemented in the frequency domain by cropping out the low frequencies. The respective upsampling operation is thus implemented by inserting zeros at the missing high frequencies. In our implementations, we combine the Gaussian profile and the downsampling operator into a single filter in the frequency domain for computational efficiency. As the Gaussian profile is performed in a manner of a low-pass filter, truncating high frequencies for downsampling does not cause intensity oscillations in the image domain.

The spatial image gradient guides the HR reconstruction. The index set S in the regularization term of Equation (3) comprises 40 spatial gradient fields that yield different orientations and different scales, as suggested in Sui et al. (2019). All the 40 gradient fields are combined into a gradient guidance, denoted by **g** in Equation (3). The *s*-th component of **g** is separately computed from the image constructed by the interpolation and average (IAA) method. In the IAA method, the *n* aligned LR images are interpolated to the same size at the same resolution as the HR reconstruction, and then the reconstructed HR image is formed by averaging out the *n* interpolated images. Specifically, with an image obtained by IAA, denoted by *I*, a component of the gradient guidance is calculated by $I-D_{x}^{\alpha }D_{y}^{\beta }D_{z}^{\gamma }I$ where $D_{m}^{n}$ denotes the operation that circularly shifts an image in *m*∈{*x, y, z*} direction by *n* voxels. We set α to integers between −2 and 2, and β and γ between 0 and 2. We exclude the components calculated at α = β = γ = 0 and α+β+γ <0 to eliminate the replicates. Consequently, we have 40 components calculated for the gradient guidance. We put all the 40 components in a set S and index them by **g**_*s*_ in Equation (3). We set the regularization weight parameter λ in Equation (3) to 0.1 in all experiments in this paper according to our experimental investigation.

The source codes and a docker version of our reconstruction algorithm can be checked out from our website^3^.

### 2.3. Assessment Criteria

We assess our approach in terms of spatial resolution, SNR, CNR, and acquisition time.

#### 2.3.1. Spatial Resolution

The signal intensity in a voxel is quantified as the integration of the signal over a spatial region defined by the position and size of the voxel. Spatial resolution is usually used to describe in an image the number of *independent* voxels per unit length or volume. Different from the measure based on voxel size, spatial resolution refers to the ability to differentiate two types of brain tissues that are relatively close together. As partial voluming artifacts occur due to the dependent voxels, the number of voxels suffering from partial volume effects can be an effective measure for spatial resolution. The higher the spatial resolution, the fewer the voxels affected by partial volume effects. To this end, we evaluate the percentage of the voxels that comprise the signal from more than one type of brain tissue and use it as the metric of the partial volume effect estimation. An image at a higher spatial resolution thus yields a lower metric value of partial volume effect.

We consider three types of brain tissues in the estimation of the partial volume effect from the neonatal MRI reconstruction: cerebrospinal fluid (CSF), gray matter (GM), and white matter (WM). As the three tissues yield different contrasts in MR images, the intensities of the voxels from them scatter in three clusters. Due to the partial voluming, there may be overlaps in the three clusters. We thus investigate the distribution of the voxel intensities of the HR reconstruction. First, we select an image region that contains the three tissues, and then construct a histogram of the voxel intensities over the selected image region. It has been shown in Laidlaw et al. (1998) that the distribution of voxel intensities from a pure tissue is Gaussian. Therefore, we fit the histogram of the voxel intensities by a Gaussian mixture model (GMM) with three components that characterize the three types of brain tissues. The voxels from a pure tissue are thus identified if their intensities range from μ−δ to μ+δ for μ and δ being the mean and half of FWHM of the corresponding Gaussian component in the GMM, respectively. We apply the GMM to the entire image to form the voxel set-1 containing all voxels from the three tissues (i.e., voxels may contain the signal from more than one tissues) and set-2 consisting of the voxels identified from each pure tissue (i.e., voxels contain the signal from only one tissue). The difference between the two sets of voxels consequently indicates the number of voxels suffering from the partial volume averaging.

#### 2.3.2. SNR and CNR

We compute the SNR of an image from the mean of signal intensities over the noise. Specifically, the SNR is found by $SNR=10\log_{10}\frac{\overset{3}{\underset{k=1}{\sum }}w_{k}s_{k}}{\sigma \overset{3}{\underset{k=1}{\sum }}w_{k}}$ where *s*_*k*_ and *w*_*k*_ denote the mean signal intensity of the voxels and the percentage of the voxels from the *k*-th pure tissue, respectively, and σ denotes noise measure. Both *s*_*k*_ and *w*_*k*_ can be directly obtained from the fitted GMM constructed above. *s*_*k*_ is computed from the mean of the *k*-th Gaussian component, while *w*_*k*_ is evaluated as the maximum of the *k*-th Gaussian component. We select an image region in the background and compute the standard deviation of the voxel intensities over the region as the noise measure.

Similar to SNR, we compute the CNR from the difference of the mean of signal intensities between two types of tissues over the noise: $CNR_{j,k}=10\log_{10}\frac{\left|s_{i}-sj\right|}{\sigma }$ We evaluate in the assessment the CNR between CSF and GM, denoted by CNR:CSF-GM, the CNR between CSF and WM, denoted by CNR:CSF-WM, and the CNR between GM and WM, denoted by CNR:GM-WM.

### 2.4. Experimental Design

We conduct two experiments to assess our approach on simulated data as well as the data acquired from 20 newborns on a Siemens 3T scanner. The goal of the experiments is to demonstrate that our approach achieves high-quality neonatal brain MRI with reduced imaging time, which allows for the studies with both research and clinical purposes.

We leveraged two other acquisition strategies as baseline schemes to compare to in the experiments, including direct HR acquisition (DA) and the single image-based super-resolution (SISR) method. Our approach was assessed by comparing to DA to verify the improved image quality and reduced acquisition time. The SISR used the same SRR algorithm as our approach with the same parameters setting. It is in fact a special case of our approach when only one LR scan was acquired, i.e., *n* = 1 in Equation (3). We used approximately three times more slices in a single LR image than in an LR image in our approach, in order to ensure equal acquisition time (by conducting the same number of phase encoding steps) for a fair comparison. Consequently, the comparisons to SISR evaluated the superiority of our approach to variable slice selection direction over the acquisition with constant slice selection direction.

We employed other four state-of-the-art SRR methods as baseline methods to assess our approach, including the interpolation and average (IAA) method, total variation (TV) prior (Plenge et al., 2012), non-local upsampling (NLU) method (Manjón et al., 2010), and a deep convolutional network-based SRR (SRCNN) method (Dong et al., 2016). The IAA method interpolated the *n* LR images to the same size at the same resolution as the HR reconstruction by a third-order B-spline method, and then aligned all the interpolated images together. The reconstruction was finally obtained by averaging all the interpolated and aligned images. IAA is one of the most widely used methods in both clinical practices and scientific research studies due to its effectiveness in improving SNR. We therefore compared our approach to IAA to assess the applicability of our approach in practical imaging tasks. The NLU method further processed the results generated by IAA with a non-local mean algorithm. The TV method used the same deconvolution scheme as our approach to reconstruct the HR image. Our scan strategy allows for training deep 2D SRR models as it acquires in-plane HR slices. The deep SRR models can be trained on these HR slices and then used to super-resolve the through-plane LR slices to generate an isotropic HR image. Although recent years have witnessed the extensively proposed deep neural networks-based SRR methods, only lightweight deep architectures allow for the training due to the limited number of HR slices acquired with our scan strategy. We therefore employed SRCNN as a deep baseline model in the experiments, which comprises about 8 k parameters to train. The trained model was applied to the through-plane LR slices of each LR image. The reconstructed HR image was formed by averaging out all the super-resolved images on their voxels. We set the weight parameter of the TV method at 0.1 for its best results according to the simulation results. We set the parameters in NLU according to the recommendation in Manjón et al. (2010).

#### 2.4.1. Experiment 1: Simulations on MPRAGE Data

The goal of this experiment is three-fold: (1) to investigate the influence of the gradient guided regularization on the SRR performance; (2) to demonstrate that our anisotropic acquisition strategy with variable directions in slice selection leads to superior SRR to the strategy of single acquisition; and (3) to show that our SRR approach achieves the MR images of higher quality than direct HR acquisition in terms of spatial resolution and SNR.

For the experimental goal, we simulated a data set based on the Dryad data set containing eight MPRAGE images at an ultra high resolution of isotropic 250 μm (Lusebrink et al., 2017). This data set was acquired from an adult subject, and the acquisition time was about 1 h per image with very complicated protocols and pre- and post-acquisition processing operations, in order to preserve a satisfactory SNR. So it is practically impossible to acquire such images in clinical routines. As there is currently no publicly available HR neonatal brain scan, and it is challenging to acquire an HR image from a neonate at a satisfactory SNR, we used this data set for the simulation demonstrations. Considering the goal of this experiment addressed above, it is reasonable to use this data set for the demonstrations.

We generated eight images at the resolution of isotropic 0.5 mm by downsampling each original image, and used them as the direct HR acquisitions. The downsampling followed the process defined in the forward model, as shown in Equation (2). Then, we simulated three LR images based on each direct HR acquisition in the three complementary planes and used them as our anisotropic acquisitions with variable directions in slice selection. The in-plane resolution of these LR images was 0.5 × 0.5 mm and the slice thickness was 2 mm. To keep the contrast unchanged, we assumed the echo time (TE) and repetition time (TR) of these LR images the same as the direct HR acquisitions. Each direct HR acquisition comprised 193,600 phase encoding steps, while the three LR images contained 132,000 phase encoding steps in total. Therefore, the acquisition time of the three LR images was ~68% of that of the direct HR acquisition. For the single acquisition-based SRR, we generated an LR image at the resolution of 0.5 × 0.5 × 0.73 mm. The resolution was derived from that the same number of phase encoding steps (132,000 steps) were conducted for this image. All the simulations for the LR images followed the process described in Equation (2).

We investigated the regularization weight parameter λ in Equation (3) to study the influence of the gradient guided regularization on the SRR performance. We ran our SRR approach with different λ values in a certain range and evaluated the peak signal-to-noise ratio (PSNR) and structural similarity (SSIM) (Wang et al., 2004) against the ground truth image in the simulation experiment. We fixed the value of λ in all other experiments reported in this paper according to the investigation results.

We reconstructed the HR images at the resolution of isotropic 0.5 mm by using the gradient guidance regularized SRR algorithm, as shown in Equation (3) on the data sets simulated from the anisotropic and single acquisition strategies, respectively. We compared the HR images reconstructed by our approach to the HR reconstructions by SISR and the direct HR acquisitions in terms of the spatial resolution, SNR, and CNR. Through the comparisons, we can answer the questions: (1) can SRR constructs images of higher quality with lower acquisition time than direct HR acquisition? and (2) with the same acquisition time, which acquisition strategy leads to better SRR, our anisotropic acquisition or the single acquisition? The second question is essentially about how we allocate data acquisitions for a better SRR given a fixed acquisition time.

#### 2.4.2. Experiment 2: Assessment on Clinical T2 FSE Data

The objective of this experiment is to evaluate our approach on the clinical data and to demonstrate that our approach can provide high quality images for both scientific research studies and clinical routines in neonatal brains. To this end, we acquired a data set with the protocol presented above. The data set comprised 60 neonatal brain MR images acquired from 20 newborns (acquired three from each). All scans were performed in accordance with the local institutional review board (IRB) protocol. We incorporated the IAA method as a baseline in this experiment, which is one of the most widely used methods in both clinical practices and scientific research studies. The HR images were reconstructed at the resolution of isotropic 0.39 mm in this experiment by our approach and the IAA method. These reconstructed HR images were assessed in terms of spatial resolution, SNR, and CNR.

## 3. Results

Our reconstruction algorithm was implemented in MATLAB (The MathWorks Inc.) without any code optimizations. We carried out our algorithm on a workstation with an Intel Xeon CPU@2.1 GHz and 128 GB memory. It took about 15 min to reconstruct an image of the typical size 384 × 384 × 384 voxels.

We reported and visualized our quantitative results by using the box and whisker plot (McGill et al., 1978; Langford, 2006). On each box, the central mark indicated the median, and the bottom and top edges of the box indicated the 25th and 75th percentiles, respectively. The whiskers extended to the most extreme data points.

### 3.1. Experiment 1: Simulations on MPRAGE Data

**Figure 1:**
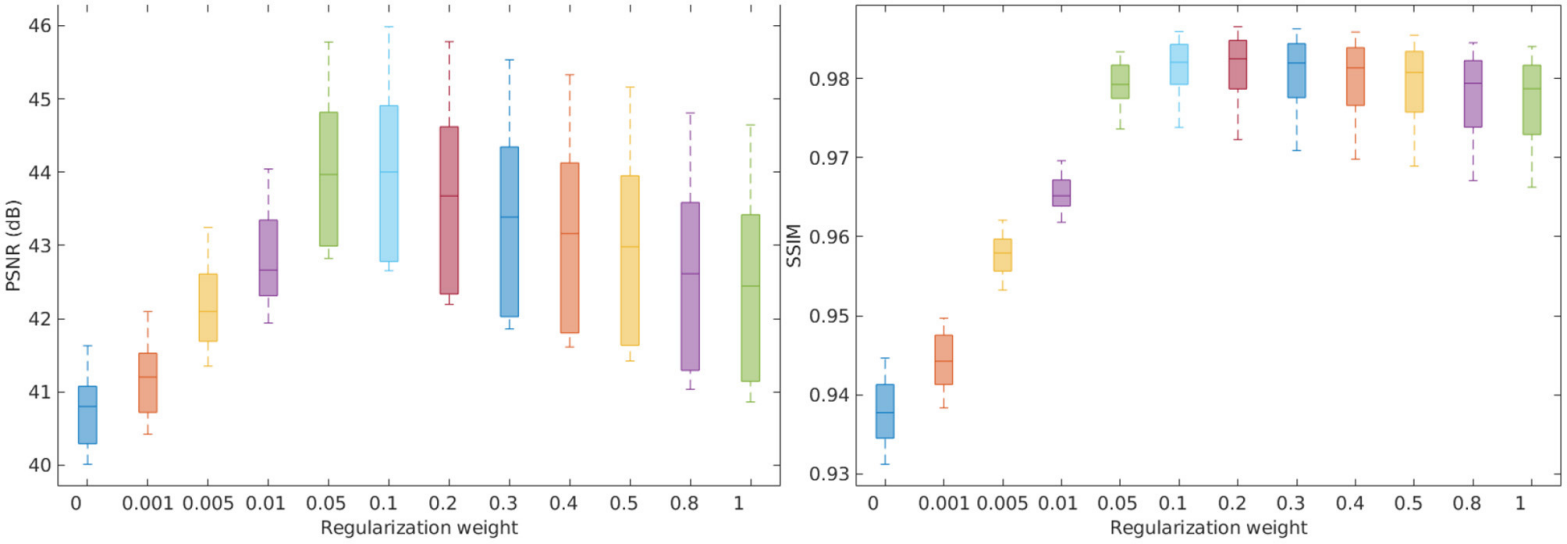
Investigation on the influence of the gradient guided regularization to the SRR performance in terms of PSNR and SSIM. The results show that our SRR approach performed the best with the regularization weight parameter λ ranging from 0.05 to 0.3. The results also suggest that the regularization considerably improved the SRR performance on the simulation data set by referring to the results at λ = 0 (in the case of no regularization).

Figure 1 shows the investigation results on the influence of the gradient guided regularization on the SRR performance in terms of PSNR and SSIM. The results show that our SRR approach performed the best with the regularization weight parameter λ ranging from 0.05 to 0.3. The results also suggest that the regularization considerably improved the SRR performance on the simulation data set by referring to the results at λ = 0 (in the case of no regularization). According to the investigation results, we therefore fixed the regularization weight parameter λ at 0.1 in all the rest experiments reported in this paper.

**Figure 2:**
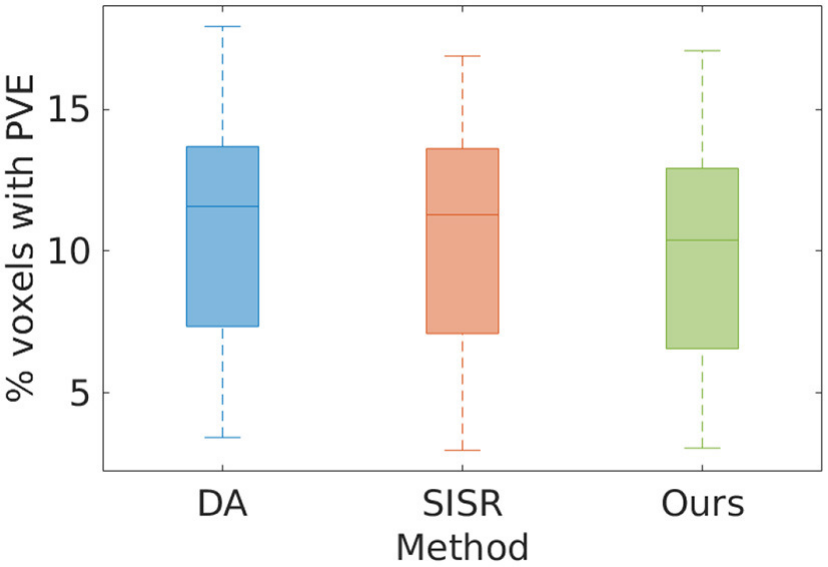
Estimates of partial volume effects (PVE) from the eight MPRAGE images directly acquired and reconstructed by SISR and our approach on the simulated data set, respectively. The average percentages of the voxels suffering from PVE were, respectively 10.82 ± 5.02, 10.48 ± 4.93, and 9.99 ± 4.80% with the methods of direct acquisition (DA), SISR, and ours. The results show that our approach generated the highest spatial resolution on this data set. Our approach yielded a 7.7% reduction in the partial volume effects, leading to the enhancement in spatial resolution, as compared to the direct HR acquisitions. The results also suggest that SRR (both SISR and our approach) achieved higher spatial resolution with much lower acquisition time than direct HR acquisition.

Figure 2 shows the estimates of partial volume effect from the eight MPRAGE images directly acquired and reconstructed by SISR and our approach on the simulated data set, respectively. The average percentages of the voxels suffering from partial volume effect were respectively 10.82 ± 5.02%, 10.48 ± 4.93%, and 9.99 ± 4.80% with the methods of direct acquisitions, SISR, and ours. The results show that our approach generated the highest spatial resolution on this data set. Our approach yielded a 7.7% reduction in the partial volume effects, leading to the enhancement in spatial resolution, as compared to the direct acquisitions the resolution of isotropic 0.5 mm. The results also suggest that SRR (both SISR and our approach) achieved higher spatial resolution with much lower acquisition time than direct HR acquisition.

**Figure 3:**
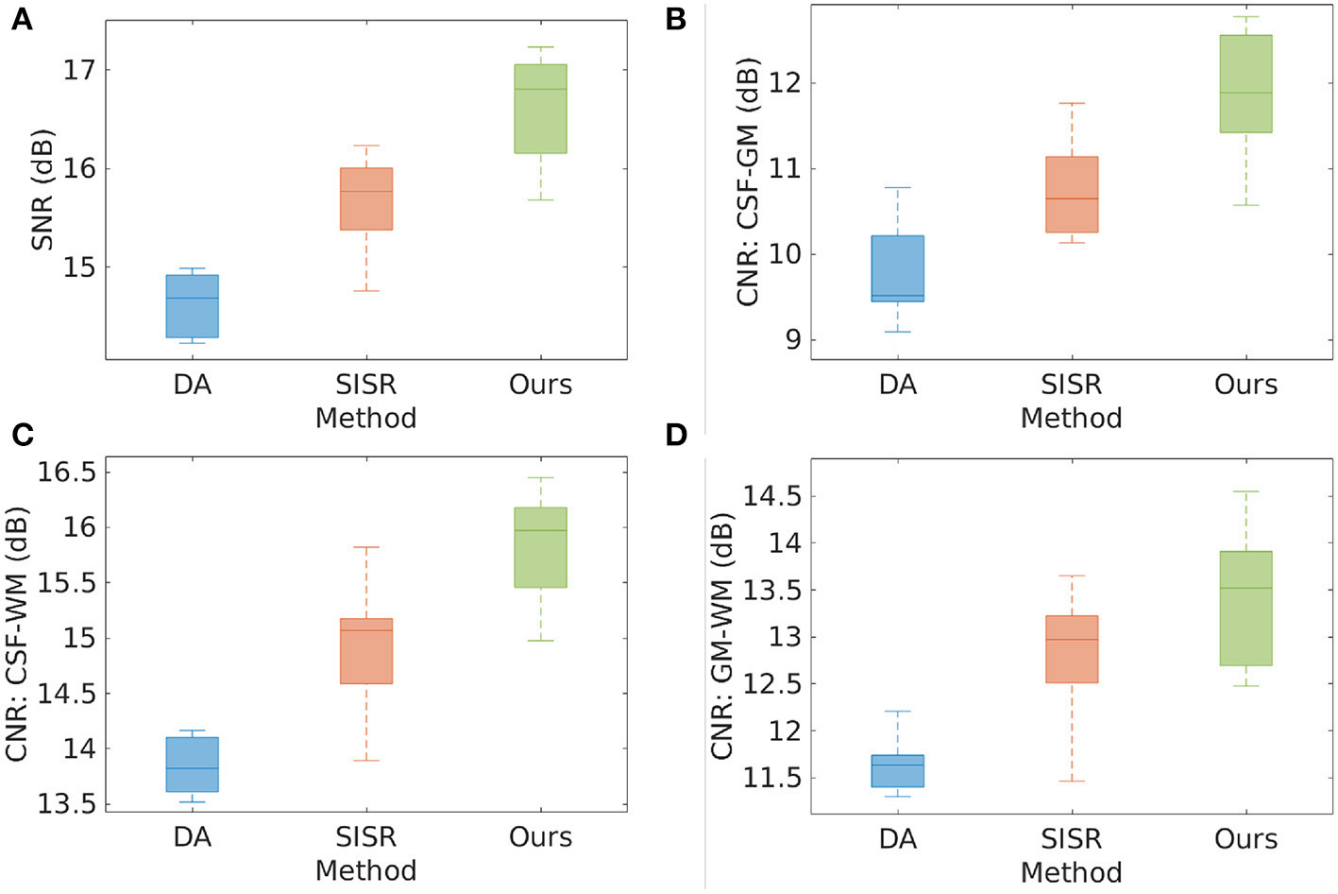
Results of direct acquisition (DA), SISR, and our approach in terms of SNR and CNR from the eight MPRAGE acquisitions/reconstructions from the simulated data set. **(A)** The average SNRs obtained from DA, SISR, and our approach were 14.51 ± 0.57, 15.66 ± 0.48, and 16.62 ± 0.56 dB, respectively. Our approach achieved higher SNR on this data set, and yielded 2.11 dB enhancement in SNR as compared to direct acquisition. Two-sample *t*-test at the 5% significance level showed that our approach significantly outperformed DA (*p* = 3.02*e*^−6^) and SISR (*p* = 2.40*e*^−3^). Wilcoxon signed-rank tests, where the null hypothesis was the difference of two sets of data comes from a distribution with zero median, showed that the population mean rank of our approach significantly differed from the two baselines in SNR at the 5% significance level (rejected the null hypothesis with *p* = 7.8*e*^−3^ for both DA and SISR). **(B–D)** Our approach consistently offered the highest CNRs between the three types of brain tissues on this data set. In particular, our approach achieved 1.31 dB higher CNR between GM and WM than direct acquisition. The results show that SRR led to considerably improved SNR and CNR as compared to direct HR acquisition.

Figure 3 shows the results of direct acquisition, SISR, and our approach in terms of SNR and CNR from the eight MPRAGE acquisitions/reconstructions on the simulated data set. The average SNRs obtained from direct acquisition, SISR, and our approach were 14.51 ± 0.57, 15.66 ± 0.48, and 16.62 ± 0.56 dB, respectively. Our approach achieved higher SNR on this data set, and yielded 2.11 dB enhancement in SNR as compared to the direct acquisitions. Two-sample *t*-test at the 5% significance level showed that our approach significantly outperformed DA (*p* = 3.02*e*^−6^) and SISR (*p* = 2.40*e*^−3^). Wilcoxon signed-rank tests, where the null hypothesis was the difference of two sets of data comes from a distribution with zero median, showed that the population mean rank of our approach significantly differed from the two baselines in SNR at the 5% significance level (rejected the null hypothesis with *p* = 7.8*e*^−3^ for both DA and SISR). Our approach consistently offered the highest CNRs between the three types of brain tissues on this data set. In particular, our approach achieved 1.31 dB higher CNR between GM and WM than direct acquisition. The results show that SRR led to considerably improved SNR and CNR as compared to direct HR acquisition.

**Figure 4:**
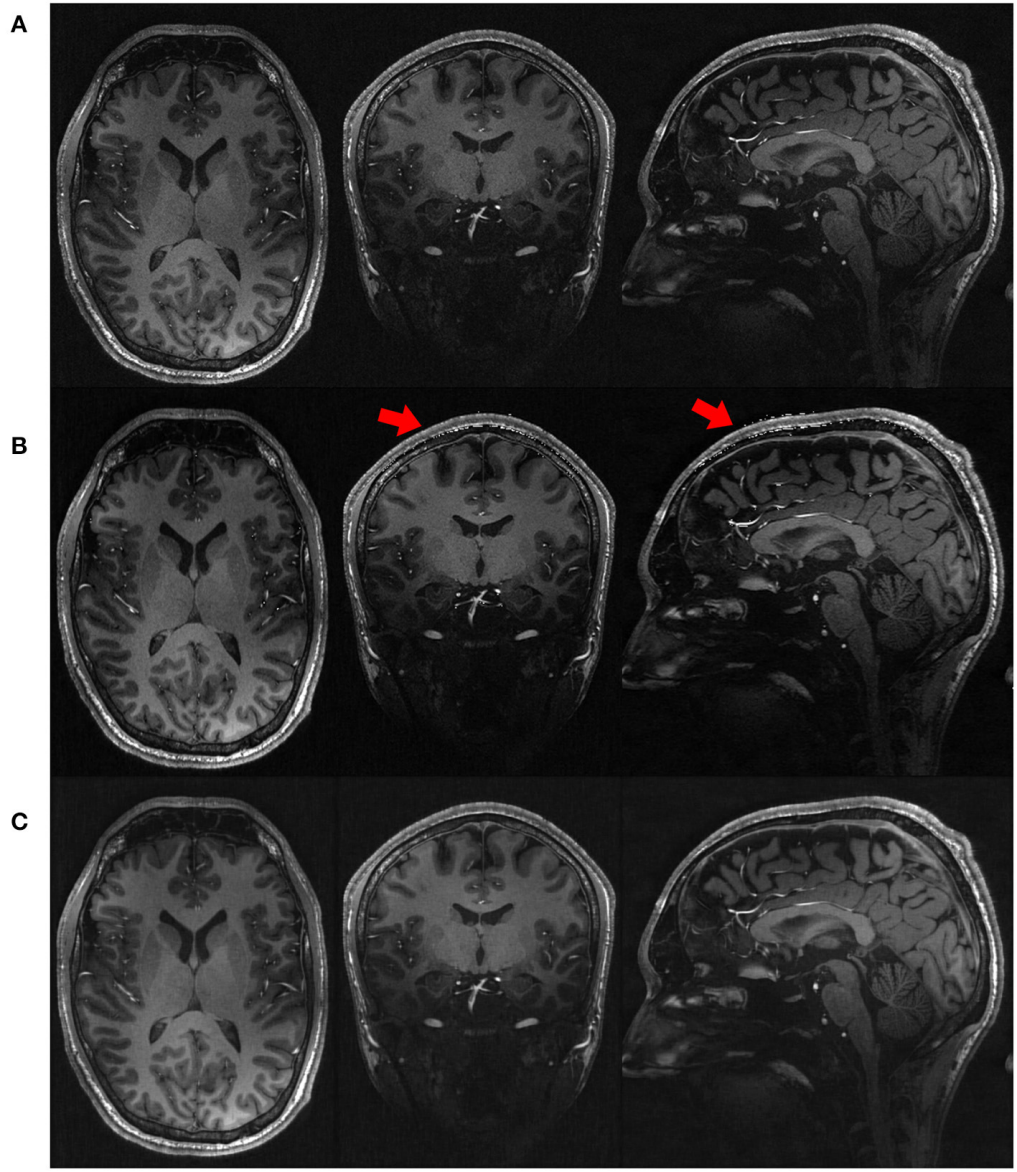
Qualitative results in representative slices from the images **(A)** directly acquired, **(B)** reconstructed by SISR, and **(C)** reconstructed by our approach, respectively, on the simulated data set. The slices directly acquired and formed by SISR were much noisy as compared to our reconstructions. The noise was more prominent for SISR in the voxels from the skull, as highlighted by the red arrows. Although what we were interested in were CSF, GM, and WM, the noisy voxels from the skull rendered that SISR generated noise all over the images but just not as obvious as those from the skull.

Figure 4 shows the qualitative results in representative slices from the images directly acquired, reconstructed by SISR and our approach, respectively. The slices directly acquired and formed by SISR were much noisy as compared to our reconstructions. The noise was more prominent for SISR in the voxels from the skull, as highlighted by the red arrows. Although what we were interested in were CSF, GM, and WM, the noisy voxels from the skull rendered that SISR generated noise all over the images but just not as obvious as those from the skull.

### 3.2. Experiment 2: Assessment on Clinical T2 FSE Data

**Figure 5:**
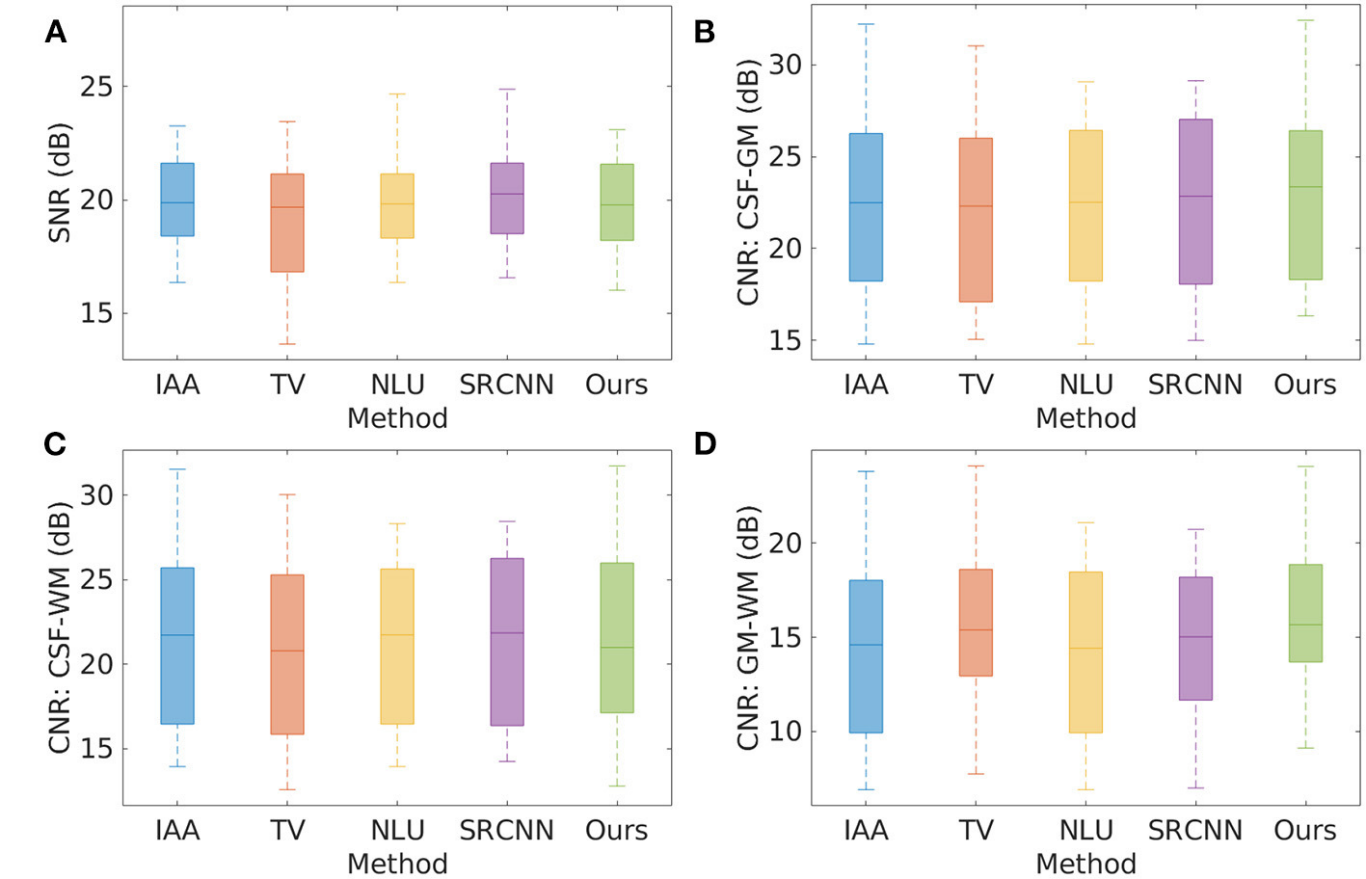
Quality of the 20 HR images reconstructed by the five SRR methods on the clinical data set in terms of SNR and CNR. **(A)** SNR; **(B)** CNR between cerebrospinal fluid and gray matter; **(C)** CNR between cerebrospinal fluid and white matter; and **(D)** CNR between gray matter and white matter. The average SNR achieved by the five methods are, respectively: IAA = 20.19 ± 2.57 dB, TV = 19.17 ± 3.40 dB, NLU = 19.92 ± 2.04 dB, SRCNN = 20.18 ± 1.98 dB, Ours = 20.04 ± 2.77 dB. IAA, NLU, and SRCNN generated high SNR, as they benefited from the averaging to improve the SNR and CNR. Our approach offered comparable SNR with IAA, NLU, and SRCNN, and outperformed TV by ~1 dB in terms of SNR. Our approach generated slightly superior CNRs to the five baselines about cerebrospinal fluid, and yielded considerably higher CNR between gray matter and white matter than these baselines.

Figure 5 shows the quality of the 20 HR images reconstructed by the five SRR methods on the clinical data set in terms of SNR and CNR. The average SNR achieved by the five methods are, respectively: IAA = 20.19 ± 2.57 dB, TV = 19.17 ± 3.40 dB, NLU = 19.92 ± 2.04 dB, SRCNN = 20.18 ± 1.98 dB, Ours = 20.04 ± 2.77 dB. IAA, NLU, and SRCNN generated high SNR, as they benefited from the averaging to improve the SNR and CNR. Our approach offered comparable SNR with IAA, NLU, and SRCNN, and outperformed TV by ~1 dB in terms of SNR. Our approach generated slightly superior CNRs to the five baselines about cerebrospinal fluid, and yielded considerably higher CNR between gray matter and white matter than these baselines.

**Figure 6:**
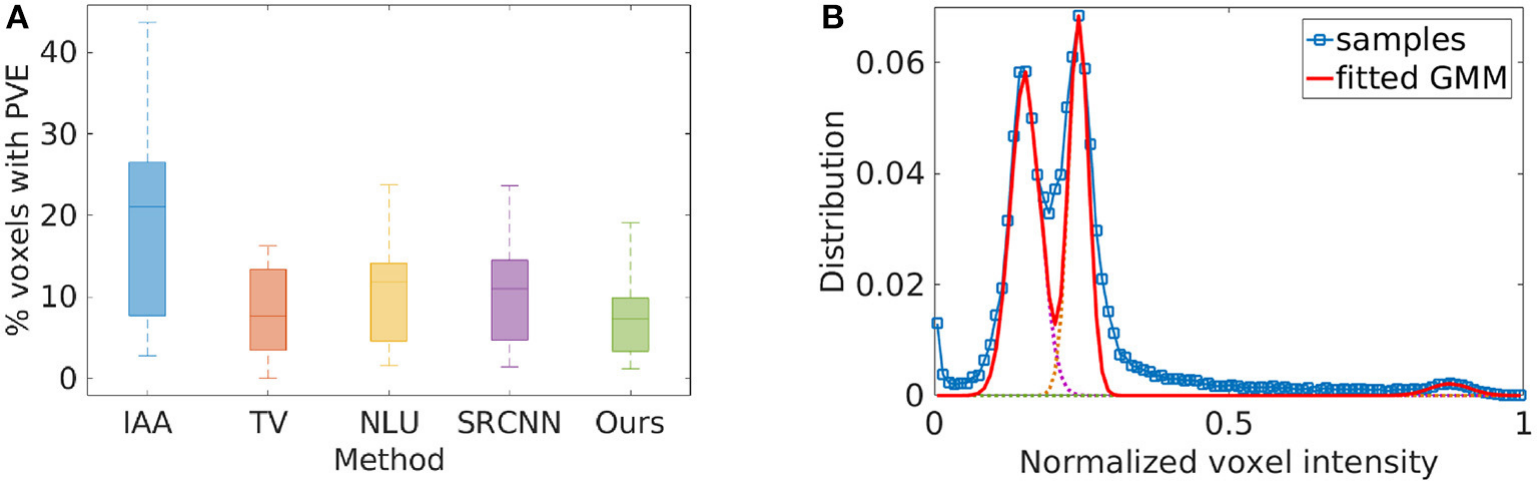
Spatial resolution evaluated from the twenty images reconstructed by the five SRR methods on the clinical data set in terms of partial volume effect (PVE). **(A)** The average PVE achieved by the five methods are, respectively: IAA = 19.40 ± 11.85%, TV = 9.02 ± 7.30%, NLU = 11.35 ± 7.69%, SRCNN =10.88 ± 7.46%, Ours = 7.25 ± 4.37%. Our approach offered a considerably lower percentage of the voxels suffering from PVE in the HR reconstructions than the four baselines, leading to substantially enhanced spatial resolution. Two-sample *t*-test at the 5% significance level showed that our approach significantly outperformed IAA (*p* = 1.40*e*^−6^), NLU (*p* = 2.65*e*^−4^), and SRCNN (*p* = 5.39*e*^−4^). Wilcoxon signed-rank tests, where the null hypothesis was the difference of two sets of data comes from a distribution with zero median, showed that the population mean rank of our approach significantly differed from the baselines in PVE at the 5% significance level (rejected the null hypothesis with *p* = 8.86*e*^−5^ for IAA, *p* = 2.76*e*^−2^ for TV, *p* = 1.89*e*^−4^ for NLU, and *p* = 2.93*e*^−4^ for SRCNN). **(B)** The demonstration of the PVE estimation on a representative image. The curve with a square marker shows the voxel distribution of the image. The dotted lines depict the three Gaussian components in the GMM. The solid line addresses the fitted GMM. The three components from left to right represented the voxels from GM, WM, and CSF, respectively. The difference in the area under the curve between the voxel distribution and the fitted GMM in the range between two successive components corresponded to the estimate of the PVE.

Figure 6 the spatial resolution evaluated from the twenty images reconstructed by the four baselines and our approach on the clinical data set in terms of partial volume effect. The average PVE achieved by the five methods are, respectively: IAA = 19.40 ± 11.85%, TV = 9.02 ± 7.30%, NLU = 11.35 ± 7.69%, SRCNN = 10.88 ± 7.46%, Ours = 7.25 ± 4.37%. Our approach offered a considerably lower percentage of the voxels suffering from partial volume averaging in the HR reconstructions than the four baselines, leading to substantially enhanced spatial resolution. Two-sample *t*-test at the 5% significance level showed that our approach significantly outperformed IAA (*p* = 1.40*e*^−6^), NLU (*p* = 2.65*e*^−4^), and SRCNN (*p* = 5.39*e*^−4^). Wilcoxon signed-rank tests, where the null hypothesis was the difference of two sets of data comes from a distribution with zero median, showed that the population mean rank of our approach significantly differed from the baselines in PVE at the 5% significance level (rejected the null hypothesis with *p* = 8.86*e*^−5^ for IAA, *p* = 2.76*e*^−2^ for TV, *p* = 1.89*e*^−4^ for NLU, and *p* = 2.93*e*^−4^ for SRCNN). Figure 6B shows the demonstration of the partial volume effect estimation on a representative image. The curve with a square marker shows the voxel distribution of the image. The dotted lines depict the three Gaussian components in the fitted GMM. The solid line addresses the fitted GMM. The three components from left to right represented the voxels from GM, WM, and CSF, respectively. The difference in the area under the curve between the voxel distribution and the fitted GMM in the range between two successive components corresponded to the estimate of the partial volume effect.

**Figure 7:**
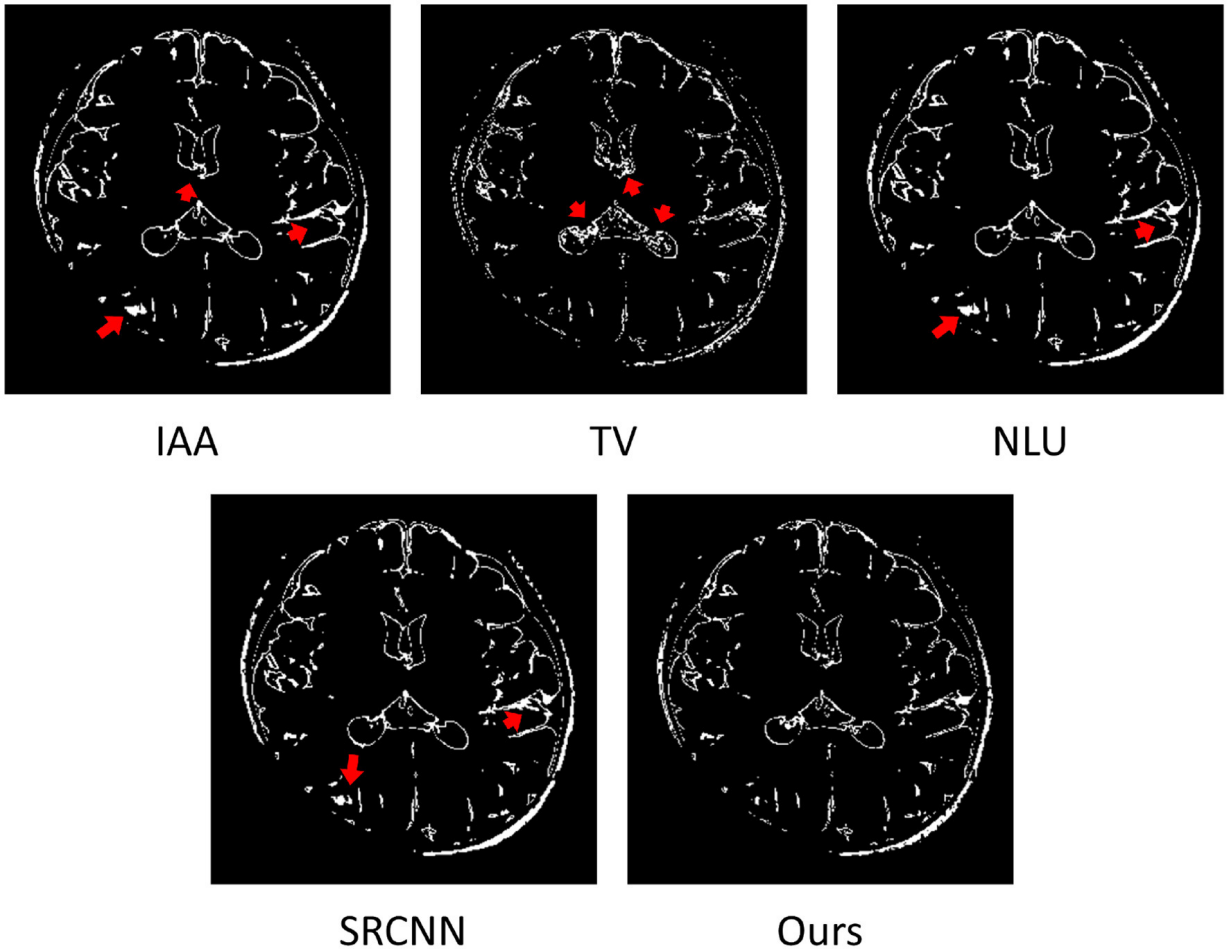
Estimated voxels suffering from partial volume effect (PVE) in the representative slice of from the image reconstructed by the four baselines and our approach. The results show that almost all voxels with PVE were from the boundaries between different types of brain tissues. Our approach comprised much fewer voxels with PVE than the four baselines. The red arrows highlight the image regions with severe PVE in the slice obtained from the four baseline methods. The results demonstrate that our approach offered considerably enhanced spatial resolution of this image.

Figure 7 shows the estimated voxels suffering from partial volume averaging in the representative slice from the image reconstructed by the four baselines and our approach, respectively. The results show that almost all voxels with partial volume effect were from the boundaries between different types of brain tissues. Our approach comprised much fewer voxels with partial volume effect than the four baseline methods. The red arrows highlight the image regions with severe partial volume effect in the slice obtained from the four baseline methods. The results demonstrate that our approach offered considerably enhanced spatial resolution of this image.

**Figure 8:**
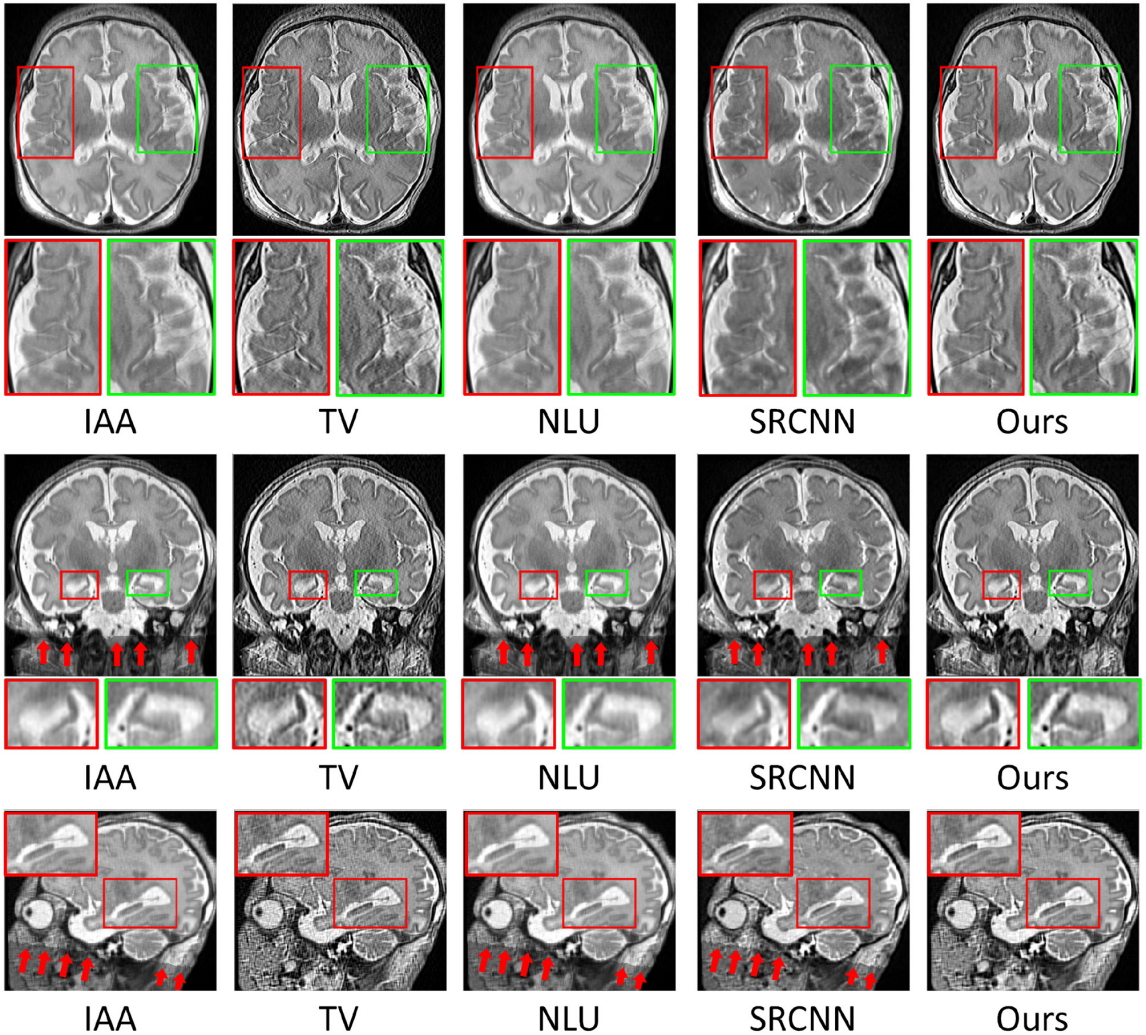
Qualitative results in representative slices of the images reconstructed by the five SRR methods. Our approach achieved the best qualitative performance with regarding to the image contrast and sharpness, in particular, on the delineation of the structures of the hippocampus as shown in the coronal and sagittal planes. The TV method sharpened the image excessively, resulting in the noisy reconstruction. Our approach appropriately suppressed the noise contamination while enhancing the sharpness of the image edges. The images reconstructed by IAA, NLU, and SRCNN contained artifacts caused by averaging the images transformed due to the alignment, as highlighted by the red arrows. In contrast, our approach was not affected by the alignment.

Figure 8 shows the qualitative results in representative slices of the images reconstructed by the five SRR methods. The results show that our approach achieved the best qualitative performance with regarding to the image contrast and sharpness, in particular, on the delineation of the structures of the hippocampus as shown in the coronal and sagittal planes. The TV method sharpened the image excessively, resulting in noisy reconstructions. Our approach appropriately suppressed the noise contamination while enhancing the sharpness of the image edges. The images reconstructed by IAA, NLU, and SRCNN contained artifacts caused by averaging the images transformed due to the alignment, as highlighted by the red arrows. In contrast, our approach was not affected by the alignment.

## 4. Discussion

We have developed a methodology to perform fast and high-resolution neonatal brain MRI. This methodology allows for high spatial resolution, high SNR and CNR, and reduced scan time, in comparison to direct HR acquisition. We have achieved high quality brain MRI at a spatial resolution of isotropic 0.4 mm with 6 min of imaging time. We have also demonstrated our approach on simulated data as well as clinical data acquired from twenty newborns. The experimental results have demonstrated that our approach allows for fast and high-quality neonatal brain MRI for both research and clinical studies.

We have shown in the simulation experiment that SRR achieved 7.7% lower partial volume effects and 2.11 dB higher SNR than the direct acquisitions at the resolution of isotropic 0.5 mm, while, in parallel, with only 68% of scan time of direct HR acquisition, as reported in Figure 2. Because the directly acquired HR images were very noisy, the image edges were blurred by the noise and in turn the spatial resolution was reduced. In SRR, because thick slices were used, the SNR was improved in the LR images, as described in the forward model shown in Equation (2). The blur kernel **H**_*k*_ in Equation (2) reduced the noise by the low-pass filtering. The thicker the slices, the more the reduction in the noise. Furthermore, the gradient guidance regularized SRR algorithm was used to reconstruct the HR images in both SISR and our approach. This algorithm incorporates an image deconvolutional filter that allows for further noise reduction in the HR reconstructions. If the scan time can be increased, e.g., taking the rest 32% of scan time to acquire more LR images with our protocol, our approach can achieve much higher spatial resolution and SNR.

In the experiment on the clinical T2 FSE data, we have shown that our approach generated comparable SNR to the IAA, NLU, and SRCNN methods while considerably higher spatial resolution. The averaging operation in the three baseline methods improved the SNR since the noise was smoothed out by the averaging. However, the averaging unexpectedly reduced the spatial resolution since it also blurred the tissue boundaries (image edges), as shown in Figure 7. Our approach, instead of averaging the data, combined the three LR images in a deconvolution manner that simultaneously improved the spatial resolution and SNR. As addressed in the forward model shown in Equation (2), the acquired image **y**_*k*_ was degraded by the convolution with the blur kernel **H**_*k*_. In the derived inverse problem defined in Equation (3), a deconvolution operation was leveraged, as an inverse operation of the convolution with **H**_*k*_, to restore the image from the blurring. This operation is also known as deblurring. As only the kernel **H**_*k*_ was involved, the noise that was filtered out in the convolution was not restored by the deconvolution, leading to an improved SNR in the reconstructed HR image. The TV method leveraged the same deconvolution scheme as our approach. However, the TV prior sharpened the image excessively as it left the local smoothing unconsidered, resulting in noisy reconstructions.

As shown in Figure 8, the IAA, NLU, and SRCNN methods introduced the artifacts caused by averaging over the different number of voxels at the lattice because the image alignment rendered some voxels with undefined intensity values. Although the artifacts were outside the brain in this case, it would be an issue if the LR images, which only contain partial regions of the brain, are included in the SRR, leading to usable reconstructions. Benefiting from the method that we used to combine the images, our approach was not affected by the alignment and did not introduce such artifacts in the HR reconstructed images. As shown in the inverse problem defined in Equation (3) for our SRR, the deconvolution operations on each LR image are jointly combined in the data fidelity term. Furthermore, the regularization incorporates a spatial gradient guidance that constrains the HR reconstruction including local smooth regions separated by strong image edges. The ℓ_1_-minimization imposed on the regularization guarantees that the local regions are not smoothed excessively. Consequently, our SRR offered both local region smoothing for homogeneous intensities and edge enhancement for tissue boundary preservation in the reconstructed HR image and did not involve the artifacts raised by the image alignment.

Our protocol allows for acquiring a T2 FSE image at the resolution of 0.39 × 0.39 × 2 mm in 2 min. It is considerably fast for neonatal MRI to obtain an image with T_2_ contrast at the resolution of isotropic 0.39 mm in 6 min of total imaging time. As a comparison, in 6 min of imaging time, we can only directly acquire a 3D T2 SPACE image at the resolution of isotropic 1 mm on our 3T scanner. Acquiring that same data at the resolution of isotropic 0.39 mm can be carried out, but acquires about 16.9 times more data, and so requires an extended acquisition time, with lengthened phase encodes, reduced readout bandwidth per pixel, and much more demanding variable flip angle calculation for signal loss in the lengthened phase encodes. Assuming we account for only the increased number of phase encodes required, this data would require 6 × 6.57 = 39.4 min to acquire. In addition, the SNR is reduced as each voxel shifts from 1 cubic mm to 0.39^3^ cubic mm, a reduction in the signal by a factor of 16.9. In order for the HR data SNR to match the SNR of the 1 cubic mm data requires increasing the SNR by a factor of 16.9, which can be done by averaging together 16.9 × 16.9 ≈ 285 acquisitions. Consequently acquired one HR image with matched SNR would require 285 × 39.4 = 11,266 min, or slightly shorter than 8 days in the MRI scanner.

Our approach enables extensive resolution critical clinical applications due to the enhanced spatial resolution and improved SNR while in parallel at reduced imaging time. It has shown that high spatial resolution facilitates the diagnosis of brain diseases, such as epilepsy (Conlon et al., 1988), multiple sclerosis (Truyen et al., 1996), and tumor characterization (Naruse et al., 1986). Our approach has achieved an isotropic spatial resolution of 0.4 mm, which allows for the clinical routines, such as the detection of signal abnormalities due to brain injury and the measurement of biometrics for impaired brain growth (Kidokoro et al., 2013), and in turn enables new assessment tools for neonatal brain MRI. Our fast and high-resolution imaging technique can be applied to the clinical and scientific research studies in the neonatal brain, such as the prediction and prognosis of brain injury (Kidokoro et al., 2014; Haebich et al., 2019), for a better understanding of the potential pathways leading to altered brain structure and outcome in the preterm infant (Inder et al., 1999; Thompson et al., 2007).

In conclusion, we have exploited the acquisition strategy for improved SRR in neonatal brain MRI, which utilizes multiple anisotropic acquisitions with variable directions in slice selection. We have achieved neonatal brain MRI at a spatial resolution of isotropic 0.4 mm with 6 min of imaging time. We have demonstrated that our approach enabled considerably fast and high-quality neonatal brain MRI, as compared to direct HR acquisition. Extensive experimental results have shown that our approach allowed for high quality neonatal brain MRI for both scientific research and clinical studies.

## Data Availability Statement

The raw data supporting the conclusions of this article will be made available by the authors, without undue reservation.

## Ethics Statement

The studies involving human participants were reviewed and approved by Institutional Review Board, Boston Children's Hospital. Written informed consent to participate in this study was provided by the participants' legal guardian/next of kin.

## Author Contributions

YS: methodology, software, validation, formal analysis, and writing. SW: conceptualization, methodology, and supervision. AG: methodology and validation. OA: methodology, validation, and data curation. All authors contributed to the article and approved the submitted version.

## Conflict of Interest

The authors declare that the research was conducted in the absence of any commercial or financial relationships that could be construed as a potential conflict of interest.

## Footnotes

^1^The term “image” indicates a volumetric image of slice stack here and hereafter.

^2^CRKIT - Computational Radiology Kit, http://crl.med.harvard.edu/software/.

^3^We are preparing the codes and the docker file, and will make them publicly available after the paper is accepted.
